# High-Definition 4K-3D Exoscope in Spine Surgery: A Single-Center Experience and Review of the Literature

**DOI:** 10.3390/medicina60091476

**Published:** 2024-09-10

**Authors:** Niccolò Innocenti, Nicoletta Corradino, Francesco Restelli, Vittoria Maria Luisa Cojazzi, Elio Mazzapicchi, Marco Schiariti, Vincenzo Levi, Francesco Costa

**Affiliations:** Spine Neurosurgery Unit, Department of Neurosurgery, Fondazione IRCCS Istituto Neurologico Carlo Besta, 20133 Milan, Italy; niccolo.innocenti@unimi.it (N.I.);

**Keywords:** spine surgery, exoscope, modern technology

## Abstract

*Background and Objectives*: Binocular optical microscopy (OM) paved the way for a new era in brain and spine neurosurgery fields with the introduction of microsurgery. Despite its enormous contribution to modern neurosurgery, OM presents some intrinsic limitations that surgeons need to face during procedures such as prolonged non-ergonomic positions and decreased vision quality to the assistant eyepiece. To overcome these limitations, in recent years, new operative tools have been introduced, such as exoscopes. Here, we present our experience with exoscopes in spine surgery. *Materials and Methods*: In the period between January 2022 and December 2023, we gradually implemented the use of a high-definition 4K-3D exoscope (ORBEYE^TM^, Olympus, Japan) in patients undergoing spinal surgery. *Results*: A total of 243 patients underwent spine surgery with exoscope magnification (47 intradural tumors, 99 lumbar degenerative cases, 79 cervical degenerative cases, 5 dorsal calcified disk herniations, 4 dural arteriovenous fistulas (dAVFs), and 9 others). We compared this cohort with a similar cohort of patients operated in the same period using OM based on different endpoints: operating time, complication rate, and infection rate. We did not find any statistically significant difference in any of the endpoints between these two groups. *Conclusions*: In our experience, the exoscope provides a better resolution of spinal anatomy and higher quality real-time images of the surgery for the entire OR team and improves the ergonomic posture of both surgeons, without lengthening the operating time and without increasing the rate of adverse events. Prospective studies with a larger cohort of patients are needed to further validate these findings.

## 1. Introduction

A proper visualization and illumination of the operative field are vital cornerstones upon which neurosurgery has evolved over the last few decades. In an era where minimally invasive surgery is advocated, visualization tools have a key role in the success of surgery. Before the advent of surgical microscopes, surgeons used various magnifying systems mounted on spectacles or headbands. However, the real revolution has been the introduction of optical microscopes (OMs), which, by implementing lighting and the magnification of the surgical field, allowed for the better identification and dissection of neurovascular structures. It was Yasargil [1,2] who made OMs routinely used in neurosurgery, paving the way for the microneurosurgical era [3].

Surgical microscopes have undergone a long period of evolution and development, providing better and better resolution and magnification. In spinal neurosurgery, the advent of OMs in the 1970s led to huge improvements in surgical techniques and procedures. OMs have provided the possibility of introducing microsurgical instruments, thanks to a wide range of magnification and illumination of spinal anatomy, as well as 3D visualization possible due to their hydraulic counterbalance system and binocular viewing lenses [4].

Despite their enormous contribution to neurosurgery, OMs are burdened by various limitations: Notably, 3D visualization is limited to the operating and assisting surgeon, binocular lenses have limited mobility, and some models do not offer the same vision quality as the assistant eyepiece. Furthermore, OMs have usually huge dimensions, which impairs their maneuverability and mobility in the operating room (OR). Another pitfall is that surgeons typically assume non-ergonomic postures for a prolonged time, and this leads to an increased risk for musculoskeletal disorders [5,6,7].

For these reasons, in the last decade, engineers and surgeons have been elaborating new strategies to try to overcome these aspects. Starting from the 1980s, endoscopic procedures started to gain progressive popularity, particularly in Europe and Asia, aiming to obtain equivalent surgical outcomes with shorter hospital stays. Full-endoscopic spine surgery [8] offers distinct advantages, which vary based on the overall health status of the patient and the technical complexity of the planned procedure.

Between these two visualization tools, exoscopes have been more recently added to the neurosurgeons’ armamentarium as a promising alternative to traditional operative microscopes.

Exoscopes are new hybrid optical instruments that have characteristics that lie in between OMs and endoscopes. Although quite similar to an endoscope (EN), they are positioned outside (“exo”) the body cavity. Surgical exoscopes provide a longer working distance, greater magnification, and a wider field of view compared to common OMs, and they have a light-emitting diode (LED) source that results in less thermal tissue damage and tissue glare. They permit neutral body positioning for both the first and second operators during surgery regardless of viewing angle; this would not be possible with conventional microscopes. They comprise innovative tools to obtain the magnification of the surgical field consisting of 3D screens with 4K or HD resolution and a 3D camera with dedicated 3D glasses depending on the various models. The transition from OMs to digital screens changes the OR setup completely, introducing solutions as well as new critical points [9,10]. Whether the exoscope can truly act as a substitute for an OM is uncertain; however, more and more studies have been published in the literature regarding patient outcomes [10,11,12], and usability experience is therefore required to facilitate decision making for those considering the use of an exoscope in practice. In their meta-analysis, Begagić et al. found that exoscopes offer higher video quality and better ergonomic features without finding significant differences in intraoperative complications, lengths of procedures, and intraoperative blood loss [12].

Here, we describe our experience using the exoscope routinely for a variety of spinal neurosurgical cases, focusing specifically on assessing the potential differences in terms of intraoperative complications and the length of the procedures.

## 2. Materials and Methods

**Figure 1:**
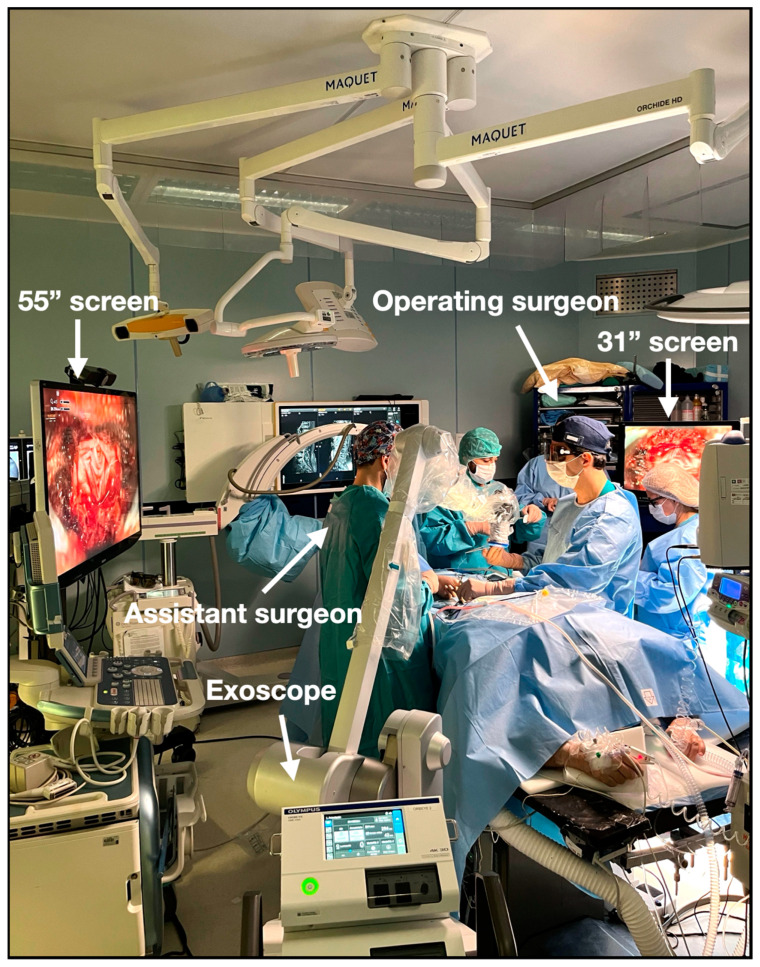
Operative room setup during the resection of a lumbar intradural tumor.

**Figure 2:**
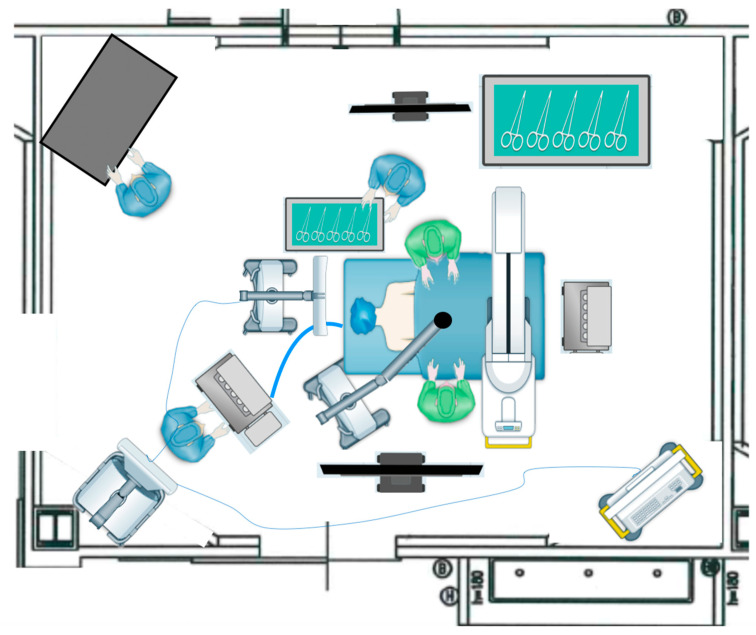
Representation of Operative room setup during spine procedure with intraoperative imaging acquisition system and navigation system.

During the period spanning from January 2022 to December 2023, we conducted a prospective analysis of all surgical procedures performed on patients diagnosed with various spinal pathologies at our institution. All the procedures were performed by the same team of spinal surgeons led by F.C., a senior spinal neurosurgeon. At our center for spinal procedures, we routinely use the Pentero^TM^ and Kinevo^TM^ microscopes (Carl Zeiss Meditec, Jena, Germany), and starting from January 2022, we implemented the use of the ORBEYE^TM^ exoscope (Olympus, Tokyo, Japan). The exoscope operative room setup involved positioning a 55″ inch 4K-3D monitor directly in front of the operating surgeon, while a second monitor of 31″ inch was placed in front of the assisting surgeon. Additionally, the exoscope itself was positioned laterally to the assisting surgeon, facilitating optimal visualization and coordination between the surgical team, as illustrated in Figure 1 and Figure 2.

In this study, different operative data were recorded and analyzed: the operating time, the complication rate, the switch from one magnification tool to the other, and the infection rate. Subsequently, we conducted an analysis comparing the outcomes between patients operated using the exoscope and patients operated using the “traditional” operative microscope (OM), analyzing them in subgroups based on specific spinal pathologies. To ensure methodological rigor and facilitate reproducibility, we focused our analysis on the most common and standardized surgical procedures performed within our series: anterior cervical discectomy and fusion (ACDF) of 1–2 levels, ACDF more than 3 levels and anterior cervical corpectomy and fusion (ACCF), lumbar microdiscectomies and one-level micro-decompression and posterior lumbar interbody fusion (PLIF) or transforaminal lumbar interbody fusion (TLIF) of 1–2 levels. We specifically focused on comparing operating time and intraoperative complications.

A direct comparison of quantitative variables was performed using an unpaired *t*-test, while Fisher’s exact test was performed for categorical values. All *p*-values were based on two-tailed tests, and differences were considered significant when *p* < 0.05.

In addition to that, a literature review on PubMed and Scopus databases was conducted searching for articles describing the use of exoscope in spinal surgery. Search terms included (exoscope OR exoscopic visualization) AND (spine surgery OR spinal surgery). Duplicate articles, articles without full text available or not written in English, clinically non-relevant studies, and case reports were excluded.

## 3. Results

Between January 2022 and December 2023, a total of 569 patients underwent spinal surgeries at our institution: 326 (57.3%) using OMs and 243 (42.7%) using ORBEYE^TM^ exoscope. In 2022, when the exoscope was first introduced, it was used in 39 out of 294 spinal surgeries (13%) at our department, while the following year, the exoscope was used in 204 out of 275 spinal surgeries (74%).

In all surgeries using ORBEYE^TM^, surgeons worked facing each other wearing 3D eyeglasses, with the first surgeon looking at the main 55” 4K-3D monitor and the assistant looking at a secondary 31″ 4K-3D monitor (Figure 1 and Figure 2).

Among the 243 patients in the exoscope group, anterior cervical discectomy and fusion (ACDF) was the most commonly performed procedure, constituting approximately 24% (59/243) of surgeries, closely followed by posterior/transforaminal lumbar interbody fusion (PLIF/TLIF), which accounted for 20.5% (50/243) of the surgeries. Intradural tumors were the third most encountered pathology, representing 19% (47/243) of the cases.

Within the lumbar region, which was the treated area in 112 patients (the most represented site in our study group, accounting for 46% of the cases), microdiscectomy and microdecompression followed the PLIF/TLIF category, each representing roughly 10% of the cases, respectively. The remaining cases comprised posterior cervical and dorsal decompression, transthoracic approach for calcified disc herniation, dural arteriovenous fistulas (dAVFs), dural tear repair, posterior dorsal arthrodesis, intramedullary dorsal cyst evacuation, and a synovial cyst resection. A detailed summary of the various surgical procedures divided based on the region of interest can be seen in Table 1.

Regarding the “OM” group, the most commonly performed surgical procedure was lumbar/lumbosacral microdiscectomy, which represented 21.4% (70/326) of the procedures, closely followed by ACDF and PLIF/TLIF, respectively, representing 19% (62/326) and 16.5% (54/326) of the cases. All the surgical operations are listed in Table 2.

Analyzing the “exoscope” group, in none of the cases was a switch to “standard operative microscopy” deemed necessary, and all surgeries were completed uneventfully without technical problems. None of the surgeons found it difficult to switch from OMs to exoscopes: Nausea, headache, and discomfort were reported neither during surgery nor at the end of the surgeries. Both the first and second surgical operators described better postural comfort within the surgery with respect to the standard OM in all cases, and the scrub assistant and the rest of the operating room staff reported excellent intraoperative vision, which was subjectively superior to the standard OM.

The mean follow-up was 16.5 mos. ± 2.3 mos. (ranging from 4 to 28 mos.) in the OM group, while it was 12.3 mos. ± 1.8 mos. (ranging from 4 to 28 mos.) in the exoscope group; the latest follow-up was in April 2024.

### 3.1. Complications

The overall incidence of surgery-related complications over the study period is detailed in Table 3. The most common complication was accidental durotomy, which occurred in 2.45% (8/245) of the cases in the microscope group and 2.05% (5/243%) of the cases in the exoscope group. A total of five surgical site infections (SSIs) occurred: two cases in the exoscope group and three cases in the microscope group. Four of the five cases were resolved with a conservative approach (inpatient IV antibiotic treatment), while in one case, surgical debridement of the site was necessary for a Klebsiella pneumonia abscess developed after a lumbar microdiscectomy. Two cases of hematomas that required surgical evacuation were observed without neurologic sequelae for the patients. We observed one case of a left C5 palsy after a single-level ACDF with an immediate post-op decrease of around 50% of left biceps brachii and left deltoid strength that slowly improved in the following months: at the 12-month follow-up, a complete recovery of the biceps brachii deficit was observed, with still a slight motor deficit in the deltoid muscle.

No statistical differences were found comparing the two groups; all *p*-values were >0.05: they were equal to 1 for SSI, accidental dural tear, and C5 palsy and equal to 0.509 for hematoma complication (Table 4).

### 3.2. Operative Time

We analyzed the mean operative time between the exoscope and the OM groups for procedures that we thought could be standardized and less dependent on the single case characteristics per se, such as for intradural tumors or long posterior fixation constructs in which a comparison could be much relevantly impacted by the intrinsic differences in the cases. We identified these “standardized” surgical operations as ACDF 1–2 levels, ACDF ≥ 3 levels or ACCF, lumbar microdiscectomy/microdecompression, and PLIF/TLIF 1–2 levels.

#### 3.2.1. ACDF 1–2 Level Subgroup

Fifty-two patients underwent single-level/double-level ACDF surgery in the exoscope group, and the mean operative time was 63.26 ± 14.9 min, while in the microscope group, fifty-nine patients underwent this procedure, with a shorter mean operative time of 56.92 ± 19.6 min. Despite the mean operative time being longer in the exoscope group, the difference was not statistically significant (*p*-value > 0.05).

#### 3.2.2. ACDF ≥ 3 Levels or ACCF Subgroup

Nineteen patients in the exoscope group and sixteen patients in the microscope group had cervical surgery with an anterior approach comprising three or more levels, or corpectomy with a mean operative time of 116.76 ± 33.2 min and 118.8 ± 65 min, respectively.

There was no significant difference in the mean operative time between the two groups (*p*-value > 0.05).

#### 3.2.3. Lumbar Microdiscectomy/Microdecompression Subgroup

One-hundred and thirteen patients underwent lumbar discectomy or single-level microdecompression in the microscope group with a mean operative time of 51.05 ± 23.8 min. A slightly shorter mean operative time was found in the exoscope group (*n* = 49): 49.57 ± 13.02 min. The difference between the two groups was not statistically significant (*p*-value > 0.05).

#### 3.2.4. PLIF/TLIF 1–2 Level Subgroup

A total of 33 patients had PLIF/TLIF surgery in the exoscope group, with a mean operative time of 143.87 ± 24.9 min, while in the microscope group, it was 140.78 ± 32.3 min among the 46 operated patients.

Despite the slightly longer operative time within the exoscope group, no statistically significant differences were found between the two groups (*p*-value > 0.05).

These results are summarized in Table 3.

### 3.3. Literature Review

As far as the literature review is concerned, 110 studies were retrieved (82 from the PubMed search and 28 from Scopus). In total, 42 studies were excluded because they were either case reports or not clinically relevant to the purpose of the present review, and 28 were excluded because they were duplicates. A total of 40 articles were included and are listed in Table S1 in Supplementary material [7,9,10,11,12,13,14,15,16,17,18,19,20,21,22,23,24,25,26,27,28,29,30,31,32,33,34,35,36,37,38,39,40,41,42,43,44,45].

## 4. Discussion

The introduction of OMs in the 1970s led to huge improvements in neurosurgery; over the years, we have seen the implementation of OMs for the magnification, illumination, and stereoscopic visualization of the surgical field. However, OMs require frequent repositioning, prolonged non-ergonomic postures, and fatigue associated with enforced focus of the operators’ eyes to the OM eyepieces. For these pitfalls, exoscopes are emerging as new visual assisting tools, providing better vision to the surgical assistant and the entire OR team and reducing postural discomfort.

Since 2010, when Mamelak et al. first described the use of a custom-made high-definition exoscope [13], the evolution of exoscope use in neurosurgery and spine surgery has been well documented. While the earliest studies established the feasibility of exoscopes [14,16], more recent ones reported excellent magnification and illumination, particularly for spinal procedures; thus, exoscopes have emerged as an alternative to microscopes for many neurosurgical procedures [12,21]. In their meta-analysis, Lei et al. reported that the use of exoscopes resulted in less intraoperative hemorrhage in anterior cervical spine surgery [30]. Overall, the literature indicates a steady progression in the acceptance and use of exoscopes in spine surgery over the past 15 years.

In our single-center experience, which covered a large spectrum of spinal procedures, the exoscope was found as a very useful tool in spine surgery. It allows for better field vision thanks to unrestricted angles of the camera, as well as ample surgical space for the use of all necessary tools such as retractors, spacers, screws, etc. Our early experience globally provides a very good impression using the exoscope. The surgical learning curve in adjusting to the exoscope magnification was fast for all surgeons, and this was also valid for experienced surgeons (who have used OMs routinely for more than 15 years), as also reported in the literature [41,45]. That is corroborated by the fact that the switch from OMs to exoscopes did not influence the mean time of surgeries or the incidence of complications in the most common and standardized spinal procedures. This value is consistent with what has been described in recent studies [12,14,43]. A comparison regarding the other surgical procedures, such as intradural tumor resection, was not performed in this study due to the bias of the different types of lesions and the relatively small size of the population, limiting a conclusive analysis. In none of our cases a switch from exoscope to OM was deemed necessary, in current literature studies a switch from exoscope to traditional microscope was described in 3.1% of cases [12].

Looking at our results, it must be underlined that, in 2022, the exoscope was used mainly during the resection of intradural tumors, and that is due to its capacity to clearly define the anatomy, especially in the deep and narrow fields of view, the tridimensionality, and the recognition of arachnoid spaces and vascularization, providing a very high resolution, which is very helpful in these pathologies. Moreover, as the image definition between the two operators was equal, we tested a real “4-hand surgery” with higher participation of the assisting surgeon in the critical moment of dissection and tumor removal [46]. When it became routinely used and also utilized in other more complex spinal procedures, we failed to find significant differences with respect to the OM, as demonstrated by the statistical analysis showing similar results. The overall impression is that the better definition can be helpful during intradural procedures and revision surgeries to better define scar tissue, as well as in deep and narrow spaces such as in ACDF or when extremely inclined angulation is required. These advantages need to be added to the more comfortable and ergonomic surgeons’ posture that exoscopes provide compared to OMs, resulting in less neck/back pain in surgeons [11,47].

**Figure 3:**
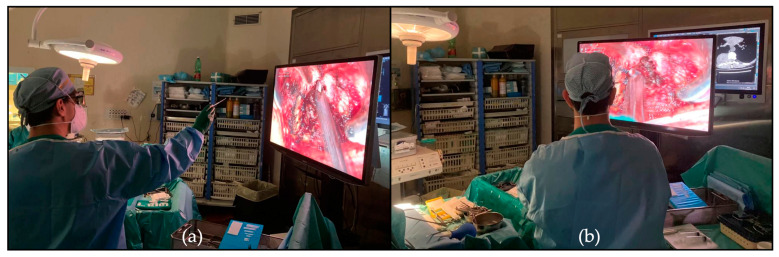
(**a**) Senior surgeon explaining the junior surgeon how to proceed during bone decompression during the resection of dorsal dumbbell schwannoma; (**b**) senior surgeon following the surgery with the same 4K-3D vision of the operating surgeon without being directly on the operating field.

Another interesting aspect is that the vision of the monitors gives all the operative room staff, including students, residents, and fellows, the possibility to share the same high-quality vision and perception of the operating surgeon, thus providing a more interactive learning experience. These features provide an immersive experience for the entire staff, helping in the anticipation of surgical steps by the team and facilitating teamwork. We found the exoscope especially valuable for surgical training; it allows for better and more interactive explanations of the surgical steps with the possibility for the senior surgeon/tutor assisting the surgery to have the same vision of the resident/junior surgeon operating without being on the surgical field (Figure 3). This would be very difficult with OMs.

However, exoscopes display some limitations. A report by Oertel and Burkhardt shows hints of the inferiority of the 3D exoscope regarding depth perception/image quality in certain circumstances [16], and this is also described by Ferreira et al. [35]. A similar result was obtained in a large controlled prospective trial by Siller et al. [9], in which, in three cases, conversion from using the 3D exoscope to OMs was performed by the attending surgeon to optimize visualization as a subjective preference. There were also reports indicating that the mechanics of the scope holder were not as easy to reposition as the hydraulic counterbalance system of the OM [9,14,15]. Siller et al. proposed a hydraulic counterbalance—for example, a robotic mechanism for the scope holder—as a possible solution to enable an easier and more precise repositioning of the 3D exoscope and improve its functionality. Another drawback is related to the operating room setup: The choice of the positioning of the monitors is crucial and may present a conflict with other devices, such as instruments used for neuronavigation, fluoroscopy, ultrasonography, etc. In the early phases, in particular, a critical aspect is the management of the area between surgeons and the monitors, as there may be an occasion in which the OR staff pass through this area, blocking the vision of the surgeons while operating. This represents another aspect of the learning curve involving not only the surgeon but all the members of the operating room, which is not well stressed in many studies.

## 5. Limitations

As this is a single-center preliminary prospective study, it presents with various limitations. The two groups “OM” and “EX” are not matched and are of different sizes, including a slightly different heterogeneous mix of procedures, and this can affect the consistency and comparability of the statistical inferences described. The considerations regarding the value of the exoscope for teaching have not been objectively measured; therefore, future studies evaluating this aspect are warranted. The lack of randomization in the decision between the use of exoscopes and OMs may result in a selection bias regarding the severity of the cases treated in the two groups.

## 6. Conclusions

High-definition 4K-3D exoscopes represent an excellent alternative to standard binocular operative microscopy (OM) in spine surgery. They overcome ergonomic and posture problems that surgeons experience with the standard OMs and, especially for the assistant, they generally provide better vision compared to the standard OMs without lengthening the operating time and without increasing the rate of adverse events. In our experience, it turned out to be a valuable tool for students/residents and fellows under training. Despite these first impressions, larger cohorts and prospective randomized studies are needed to confirm these findings and define exoscopes’ real role in spinal surgery, as well as economic studies focusing on better assessing the cost-effectiveness of this new technology.

## Figures and Tables

**Table 1:** Details of the series of 243 patients operated using ORBEYE^TM^ divided per surgical site and surgery type. ACDF = anterior cervical discectomy and fusion; ACCF = anterior cervical corpectomy and fusion; dAVF = dural arteriovenous fistula; PLIF = posterior lumbar interbody fusion, TLIF = transforaminal lumbar interbody fusion.

Surgical Site	Surgery Type (n° of Patients)
**Cervical (89)**	ACDF (59)
	ACCF (12)
	Posterior cervical decompression (8)
	Intradural/extradural tumor resection (10)
**Dorsal (38)**	Transthoracic approach for calcified disc herniation (5)
	Intradural tumor resection (24)
	Posterior decompression + fixation (5)
	dAVF (1)
	Dural tear repair (2)
	Intramedullary cyst fenestration (1)
**Lumbo-sacral (116)**	Intradural/extradural tumor resection (13)
	Microdiscectomy (24)
	Microdecompression (25)
	PLIF/TLIF (50)
	Synovial cyst resection (1)
	dAVF (3)

**Table 2:** Details of the series of 326 patients operated using “optical microscope (OM)” divided per surgical site and surgery type. ACDF = anterior cervical discectomy and fusion; ACCF = anterior cervical corpectomy and fusion; dAVF = dural arteriovenous fistula; PLIF = posterior lumbar interbody fusion, TLIF = transforaminal lumbar interbody fusion.

Surgical Site	Surgery Type (*n*° of Patients)
**Cervical (99)**	ACDF (62)
	ACCF (13)
	Posterior cervical decompression ± fixation (8)
	Intradural/extradural tumor resection (14)
	Forestier’s disease (2)
**Dorsal (34)**	Posterior decompression + fixation (16)
	Intradural/extradural tumor resection (12)
	Transthoracic approach for calcified disc herniation (2)
	Dural tear repair (2)
	Idiopathic spinal cord herniation repair (2)
	dAVF (1)
**Lumbo-sacral (193)**	Microdiscectomy (70)
	PLIF/TLIF (54)
	Microdecompression (43)
	Intradural/extradural tumor resection (22)
	Hematoma evacuation (2)
	Synovial cyst resection (1)
	Abscess evacuation (1)

**Table 3:** The incidence of operative complications over 2 years among our exoscope and microscope series. SSI = surgical site infection.

Complication type	Exoscope (%)	Microscope (%)	Overall (%)
SSI	2/243 (0.82%)	3/326 (0.92%)	5/569 (0.88%)
Accidental dural tear	5/243 (2.05%)	8/326 (2.45%)	13/569 (2.28%)
Hematoma	0/243 (0%)	2/326 (0.61%)	2/569 (0.35%)
C5 palsy	0/243 (0%)	1/326 (0.31%)	1/569 (0.18%)

**Table 4:** Comparison of mean operative time and intraoperative complications in exoscope and microscope pathologies subgroups. ACDF = anterior cervical discectomy and fusion; ACCF = anterior cervical corpectomy and fusion; dAVF = dural arteriovenous fistula; PLIF = posterior lumbar interbody fusion.

Surgery Type	Exoscope (*n*)	Mean-Operative Time (min)	Intraoperative Complications (*n*)	Microscope (*n*)	Mean-Time (min)	Intraoperative Complications (*n*)	*p*-Value Time	*p*-Value Intra-Op Compl.
ACDF 1–2 levels	52	63.26 ± 14.9	1 Surgical Site Infection	59	56.92 ± 19.6	1 (c5 palsy)	0.0741	1.00
ACDF ≥ 3 levels or ACCF	19	116.76 ± 33.2	0	16	118.8 ± 65	1 Surgical Site infection	0.9099	1.00
Lumbar Microdiscectomy/Microdecompression	49	49.57 ±13.02	2 (Accidental durotomy)	113	51.05 ± 23.8	5 (4 Accidental durotomy; 1 hematoma)	0.6851	1.00
PLIF/TLIF 1–2 levels	33	143.87 ± 24.9	3 (Accidental durotomy)	46	140.78 ± 32.3	4 (Accidental durotomy)	0.6511	1.00

## Data Availability

The original contributions presented in this study are included in the article; further inquiries can be directed to the corresponding author.

## Supplementary Materials

The following supporting information can be downloaded at: https://www.mdpi.com/article/10.3390/medicina60091476/s1, Table S1. Summary of the retrieved studies for year, study design, number of cases and key findings. OM = Optical Microscope, MIS-TLIF = minimally invasive transforaminal lumbar interbody fusion.
