# Supplementary material for: High-Definition 4K-3D Exoscope in Spine Surgery: A Single-Center Experience and Review of the Literature
Source: Medicina (Kaunas). 2024 Sep 10;60(9):1476. doi: 10.3390/medicina60091476 (PMC11434260; doi:10.3390/medicina60091476)
Supplement: Supplementary file 1 [file medicina-60-01476-s001.zip › medicina-3128615-supplementary.pdf]

Table S1. Summary of the retrieved studies for year, study design, number of cases and key findings. OM= Optical Microscope, MIS-TLIF= minimally invasive transforaminal lumbar interbody fusion.

Year	Reference	Study Design	N° cases	Key Findings
2010	Mamelak et al.	Case series	6	Image quality almost equal to microscope. Limitations in stereopsis and focusing
2012	Shirzadi et al.	Prospective cohort	24	No significant differences in operative time or hospital stay compared to microscope
2017	Krishnan et al.	Case series	10	Excellent magnification and illumination. Slightly longer operative times compared to microscope
2017	Oertel et al.	Case series	11	The 3D-exoscopic system is safe and effective for spinal procedures and less demanding cranial procedures and provide comfort for the surgical team. Image quality and 3D visualization were comparable with the OM
2019	Ricciardi et al.	Systematic review	66	Exoscope emerging as alternative to microscope for many neurosurgical procedures
2019	Barbagallo et al.	Case series	2	Exoscope provided 3D view similar to microscope, with better didactic capabilities
2019	Kwan et al.	Retrospective analysis	11	Excellent surgical and clinical outcomes without complications with an immersive surgical experience for all team members
2019	Muhammad et al.	Prospective cohort study	4	Exoscope is a safe tool to perform common spinal surgeries. Image quality is better than a microscope but with slightly less depth perception. Vigorous training in the laboratory may be helpful before clinical use
2020	Siller et al.	Prospective cohort	60	No significant differences in operative time, blood loss, or outcomes compared to microscope. Better ergonomics with exoscope
2020	Burkhardt et al.	Prospective case series	18	Exoscope rated equal or superior to microscope in 62% of cases for handling and 80% for anatomical structure identification
2020	Ariffin et al.	Prospective observational study	69	Short learning curve for exoscope use. Surgeons benefited from improved ergonomic posture. Complications were comparable to microscope use
2020	D'Ercole et al.	Retrospective case series	9	Good visualization and ergonomics. Unobstructed access to surgical field
2021	Montemurro et al.	Systematic review	274	Exoscope seems to be a safe alternative compared to microscope for common brain and spinal procedures
2021	Yao et al.	Retrospective cohort	21	Shorter operative time with exoscope. Comparable clinical outcomes to microscope
2021	Bai et al.	Retrospective case series	19	Significant improvement in clinical scores and radiographic outcomes. No complications. Safe and effective treatment
2021	Maurer et al.	Prospective cohort	6	High surgical satisfaction and improved ergonomics with exoscope use
2022	Lin et al.	Retrospective review	23	Exoscope provided comparable outcomes to microscope in ACDF procedures
2022	De Divitiis et al.	Case series	5	Exoscope offered excellent magnification and illumination for intradural extramedullary tumors
2022	Motov et al.	Prospective observational	17	Short learning curve for exoscope use. Improved ergonomics and resident teaching
2022	Yao et al.	Retrospective cohort	37	Comparable outcomes to microscope with potential advantages in ergonomics and teaching
2023	Lei et al.	Meta-analysis	154	Exoscope resulted in less intraoperative hemorrhage compared to other auxiliary means
2023	Iqbal et al.	Systematic review	539	Mixed results on operative times but present a viable alternative to OMs
2023	Schupper et al	Multicenter prospective cohort study	43	Surgeons reported significantly less neck and back pain ($P < 0.0001$) when using the robotic exoscope compared to conventional microscope or surgical loupes. No conversions to microscope were needed
2023	Sato et al.	Retrospective case series	11	No surgical complications related to exoscope use. It allows the surgeon to operate in an upright position without strain on the head and neck

2023	Giorgi et al.	Retrospective case series	10	Exoscope use resulted in small reduction of surgical time and blood loss compared to microscope. No intraoperative complications attributed to visualization mode. Better magnification, image definition, ergonomics, and user-friendliness reported with exoscope
2023	Lei et al.	Systematic review and meta-analysis	349	Exoscope resulted in less intraoperative hemorrhage compared to other auxiliary means
2023	Lin et al.	Retrospective cohort	47	Reduced surgical time and blood loss with exoscope. Improved ergonomics
2023	Yao et al.	Retrospective cohort	22	Shorter operation time and better early postoperative outcomes with exoscope-assisted MIS-TLIF
2024	Ferreira et al.	Systematic review	N/A	Exoscopes improved visualization, ergonomics, and surgical precision in spine surgeries. Some studies suggest inferior depth perception compared to OM. Possible higher cost of integrated systems
2024	Nawabi et al.	Retrospective review	123	Shorter operative time, less blood loss, and shorter length of stay with exoscope
2024	Calvanese et al.	Comparative series	19	Exoscope-assisted surgery comparable in safety and effectiveness to microscopic surgery for intradural extramedullary tumors
2024	Auricchio et al.	Comparative series	10	Exoscope-assisted surgery comparable to microscopic surgery for spinal dural arteriovenous fistulas
2024	Kusyk et al.	Prospective cohort	18	Significantly less time in deviated posture with exoscope compared to microscope
2024	Cunningham et al.	Retrospective review	96	Acceptable operative times and outcomes in pediatric spinal surgeries
2024	Vattipally et al.	Systematic Review	481	Exoscopes have advantages compared with OMs or endoscopes during spine surgery. The user learning curve is minimal, and no negative patient outcomes have been reported. Some aspects need longer-term research
2024	Das et al.	Comparative study	45	Spine surgeons can embrace the benefits of exoscope and increase their range of surgeries to be performed at small operation theater setups in low-middle income developing countries. Image quality was comparable or superior to OM in 62%-67% of cases. Preparation and installation of exoscope was much easier in 93%-100% of cases
2024	Begagić et al.	Systematic review and meta-analysis	505	Exoscopes offer superior video quality and favorable ergonomic features compared to conventional microscopes
2024	Cho et al	Technical note and retrospective review	15	Excellent clinical outcomes achieved using three-dimensional exoscopes with comparable operative time and blood loss to conventional surgical microscopes or loupes. Improved ergonomic and teaching potential noted
2024	Yamane et al.	Retrospective cohort	17	Lower postoperative neck pain scores with exoscopic minimally invasive laminoplasty
2024	Milani et al.	Retrospective cohort	22	Shorter operative time with exoscope. Comparable clinical outcomes to microscope, low learning curve for experienced neurosurgeons
